# Low rectus femoris mass index is closely associated with diabetic peripheral neuropathy

**DOI:** 10.3389/fendo.2023.1148093

**Published:** 2023-04-21

**Authors:** Lina Wang, Xiaopu Lin, Haishan Huang, Yanfang Wang, Xinxin Liang, Xiaobin Zheng, Lingling Xu

**Affiliations:** ^1^ Department of Endocrinology, Shenzhen Hospital, Southern Medical University, Shenzhen, China; ^2^ The Third School of Clinical Medicine, Southern Medical University, Guangzhou, China; ^3^ Department of Huiqiao Medical Centre, Nanfang Hospital, Southern Medical University, Guangzhou, China

**Keywords:** diabetic peripheral neuropathy, sarcopenia, rectus femoris cross-sectional area, rectus femoris mass index, ultrasound

## Abstract

**Aims:**

To assess the association of rectus femoris mass index (RFMI) with diabetic peripheral neuropathy (DPN) in individuals with type 2 diabetes mellitus (T2DM).

**Methods:**

Totally 948 T2DM cases were enrolled. Nerve conduction parameters, quantitative sensory threshold and rectus femoris cross-sectional area (RFCSA) were obtained, and rectus femoris mass index (RFMI=RFCSA/height^2^) was derived. The patients were assigned to four groups based on interquartile spacing of RFMI.

**Results:**

Motor/sensory nerve amplitude and conduction velocity (CV) were significantly lower in the low-level RFMI groups (all P<0.05). RFMI was positively associated with mean motor/sensory nerve amplitude and CV (both P<0.05). T2DM duration above 10 years and RFMI below 2.37cm²/m² had significant associations with DPN (both P<0.001). Receiver operating characteristic (ROC) curve analysis demonstrated cutoffs for T2DM duration and RFMI of 7 years and 2.2 cm²/m², respectively (AUC=0.75, 95% CI: 0.72-0.79; sensitivity, 68.4%; specificity, 66.8%).

**Conclusion:**

DPN is significantly associated with reduced RFMI in T2DM patients. Decreased muscle mass seems to be associated with motor/sensory nerve amplitude and CV. RFMI combined with T2DM duration may represent a potent tool for predicting DPN occurrence in T2DM cases.

**Clinical trial registration:**

http://www.chictr.org.cn, identifier ChiCTR2100049150.

## Introduction

Diabetic peripheral neuropathy (DPN) constitutes the commonest chronic complication of diabetes (1, 2), which affects half of patients with type 2 diabetes mellitus (T2DM) (2) and represents a major risk factor for lower limb amputation and foot ulcers (3, 4). DPN has a high incidence and insidiously onset, but its pathological severity often does not match the clinical symptoms. Totally 30-40% of patients show no clinical symptoms for a long time (5). Currently, clinical treatment strategies for DPN are limited, and efficacy is not satisfactory. Early diagnosis, screening and control of the risk factors associated with DPN is of critical clinical importance.

Sarcopenia features severely decreased skeletal muscle mass and strength (6), which is commonly found in T2DM patients. Many studies demonstrated sarcopenia prevalence is markedly elevated in T2DM cases compared with non-diabetics (7–9). DPN is associated with sarcopenia, which can result in accelerated muscle atrophy of distal lower extremity (10) and might independently predict sarcopenia (11, 12).

Measurement of muscle mass is a key factor in early screening of sarcopenia (13). Ultrasound measurement of the rectus femoris has been widely used to evaluate total body skeletal muscle mass. Mueller and collaborators demonstrated the rectus femoris muscle cross-sectional area (RFCSA) determined by ultrasound might be a quick and convenient indicator of sarcopenia (14). During muscle mass quantitation, adjustment should be made based on body shape. Height², body weight and BMI have been used (15). Height² correction of skeletal muscle mass index was first performed by Baumgartner and colleagues (16). Since then, multiple reports have utilized this index to define sarcopenia (17–20). In this study, RFCSA measured by ultrasound adjusted by height² was defined as the rectus femoris mass index (RFMI). The aim of this work was to examine the association of RFMI with DPN and to clarify whether RFMI and other indexes might predict the progression of DPN.

## Materials and methods

### Study design and patients

This cross-sectional study had approval from the Medical Ethics Committee, ShenZhen Hospital, Southern Medical University (NYSZYYEC20200030). Totally 948 T2DM patients admitted to the Department of Endocrinology, Shenzhen Hospital, Southern Medical University in April-August 2022 were enrolled. T2DM diagnosis was based on the 1999 WHO diagnostic criteria (21). The patients underwent DPN screening. Diagnosis of DPN was carried out as reported in a previous work (22): (1) abnormality in at least one nerve conduction index in ≥2 peripheral nerves; (2) at least two quantitative sensory threshold abnormalities. DPN was diagnosed when the above two criteria were met. Exclusion criteria were: (1) critical illness accompanied by ketoacidosis, hypertonic non-ketone coma, uremia and malignant tumors; (2) further neuropathy-associated parameters such as family-related factors, alcohol, nutrition, poisoning and drug addiction; (3) diseases that affect muscle metabolism, e.g., rheumatoid arthritis, poliomyelitis, Parkinson’s disease and cerebral infarction. (4) pregnancy. Signed informed consent was provided by each patient. This study has been registered in the Chinese Clinical Trials Register (ChiCTR2100049150).

### Data collection

Medical history, gender, age, height, weight, duration of diabetes and other basic indexes were collected for all patients. Body mass index (BMI) was derived as weight (kg)/height^2^ (m^2^). Systolic (SBP) and diastolic (DBP) blood pressure measurements were carried out. Laboratory data, including serum creatinine (Scr), total cholesterol (TC), triglyceride (TG), high-density lipoprotein cholesterol (HDL), low-density lipoprotein cholesterol (LDL), uric acid (UA), serum cystatin C (CysC), blood urea nitrogen (BUN), fasting plasma glucose (FPG), fasting C-peptide (FCP) and glycated hemoglobin (HbA1c) levels were assessed upon fasting for 8 h. 24-h urinary albumin excretion rate (UAER) was measured and recorded.

### Muscle ultrasound examination

RFCSA was measured on both sides by ultrasound (Philips Ultrasound, WA, USA). All measurements were taken by a single ultrasound physician. Supine-positioned patients were placed with the upper body raised by 30°, legs straight and muscles relaxed. Totally 60% of the distance from the anterior superior iliac spine to the patella’s upper edge was determined (14). An ultrasound probe was positioned perpendicularly to the thigh plane along this point to obtain a transverse image of the radio frequency. RFCSA was measured by the planar measurement technology provided by the vendor. The mean of the left and right RFCSAs was considered the final RFCSA and used for statistical analysis. RFMI (cm²/m²) was derived as RFCSA (cm²)/height² (m²).

### Nerve conduction studies (NCS)

NCS were carried out with Viking Quest. Amplitudes and conduction velocities (CVs) for the median, ulnar, tibial, and peroneal complex muscle action potential (CMAP), and sensory nerve action potential (SNAP) for the median, ulnar, common peroneal, and sural nerves were measured. Mean motor nerve amplitude was derived as: Amplitude _motor nerve_ = (Amplitude _median nerve M_ + Amplitude _ulnar nerve M_ + Amplitude _tibial nerve M_ + Amplitude _peroneal nerve M_)/4. Mean values for motor CV, sensory amplitude and sensory CV were determined similarly.

### Quantitative sensory tests (QST)

QST include the inspection of vibration perception threshold (VPT), warm threshold (WT) and cold threshold (CT). VPT, WT and CT were tested by the limit method in a standardized fashion (23). Tests were performed thrice and values were averaged. The highest value in either limb was further examined. Data were compared to the available references.

All measurements of NCS and QST parameters were performed by a single neurological technician.

### Statistical analysis

Data were analyzed with SPSS 24.0 (SPSS, USA). Normally and skewedly distributed continuous data were presented by mean ± standard deviation (SD) and median with interquartile range (IQR), respectively. Between-group comparisons utilized the Student’s t test. Multiple group comparisons applied ANOVA and the Kruskal–Wallis test for data with normal and skewed distributions, respectively. Categorical data were analyzed by χ2 test. Pearson’s correlation analysis was carried out to analyze the associations of RFMI with nerve conduction parameters (mean motor or sensory nerve amplitude and CV). Multivariable logistic regression was used to analyze associations of clinical indicators with DPN. Receiver-operating characteristic (ROC) analysis was carried out to determine the optimal cutoffs of diabetes duration and RFMI for detecting DPN. P<0.05 indicated statistical significance.

## Results

### Baseline patient features

Totally 948 diabetics aged 54.35 ± 12.71 years were included and assigned to the DPN (n=234) and non-DPN (n=714) groups (**Table 1**). DPN cases accounted for 24.7% of the overall study population. Age, gender, diabetes duration, BMI, HbA1c and RFMI were markedly different between the DPN and non-DPN groups (all P<0.001). Compared with the non-DPN group, the DPN group had elevated age, longer diabetes duration, lower BMI, higher HbA1c and lower RFMI (all P<0.001). The proportion of male in DPN group was higher compared with non-DPN group.

**Table 1:** Clinical characteristics of T2DM patients with DPN versus without DPN.

	Non-DPN (n=714)	DPN (n=234)	*P*
Age (years)	51.95 ± 12.11	61.67 ± 11.69	<0.001**
Male/female (%)	127/107 (54.3/45.7)	482/232 (67.5/32.5)	<0.001**
Duration (years)	5.00(1.00, 10.00)	10.00 (5.00, 18.50)	<0.001**
BMI (kg/m^2^)	24.83 ± 4.12	23.17 ± 4.76	<0.001**
TG (mmol/L)	1.64(1.09, 2.54)	1.51 (1.03, 2.29)	>0.05
TC (mmol/L)	4.50 ± 1.59	4.35 ± 1.35	>0.05
HDL (mmol/L)	1.15 ± 0.31	1.19 ± 0.36	>0.05
LDL (mmol/L)	2.78 ± 1.02	2.69 ± 1.10	>0.05
HbA1C (%)	9.12 ± 2.44	9.92 ± 2.52	<0.001**
RFMI	2.53 ± 0.71	2.02 ± 0.63	<0.001**

Values were expressed as mean ± SD for normally distributed data and median with interquartile range for non-normally distributed data, or n (%). Differences among the groups were analyzed by ANOVA for normally distributed values and by the Kruskal–Wallis test for nonparametric values. Pearson’s χ2 test was employed to analyze categorical data. *p<0.05, **p<0.001. BMI, body max index; TG, triglycerides; TC, total cholesterol; HDL, high-density lipoprotein; LDL, low-density lipoprotein; HbA1c, glycated hemoglobin; RFMI, rectus femoris mass index.

In addition, patients were categorized according to RFMI by interquartile range at the cut-off points of 1.96, 2.37 and 2.81 cm²/m². Patient features in various groups are shown in **Table 2**. There were significant differences in age (P<0.001), gender(P<0.001), diabetes duration (P<0.001), BMI (P<0.001), SBP (P<0.05), DBP (P<0.05), BUN (P<0.05), UA (P<0.05), CysC (P<0.001), TG (P<0.001), HDL (P<0.05), UAER (P<0.05) and DPN (P<0.001) among the groups. Age, diabetes duration, BUN, CysC, HDL and DPN increased with decreasing RFMI(P<0.05). BMI and TG declined with decreasing RFMI (both P<0.001).

**Table 2:** Clinical characteristics of T2DM patients stratified by RFMI categories.

Characteristic	RFMI				P
	>2.81 (n=238)	2.37-2.81 (n=239)	1.96-2.37 (n=231)	≤1.96 (n=240)	
Age (years)	48.33 ± 11.84	52.54 ± 11.91	56.03 ± 11.77	60.50 ± 12.10	<0.001**
Male/female (%)	183/55 (76.9/23.1)	172/67 (72.0/38.0)	144/87 (59.8/40.2)	110/130 (45.9/54.1)	<0.001**
Duration (years)	3.00 (0.46, 10.00)	6.00 (1.00, 11.00)	8.00 (2.00, 13.00)	9.00 (2.00, 115.00)	<0.001**
BMI (kg/m^2^)	26.22 ± 4.25	24.34 ± 3.30	24.21 ± 3.69	22.91 ± 5.23	<0.001**
SBP (mmHg)	128.50 ± 16.36	126.89 ± 16.03	129.75 ± 17.75	131.68 ± 20.13	<0.05*
DBP (mmHg)	81.46 ± 11.02	78.34 ± 10.59	79.10 ± 10.09	77.33 ± 10.94	<0.05*
BUN (mmol/L)	4.90 ± 1.62	5.27 ± 1.97	5.48 ± 2.49	5.49 ± 1.99	<0.05*
Cr (μmol/L)	78.51 ± 25.88	77.53 ± 25.63	79.38 ± 39.45	74.10 ± 32.98	>0.05
UA (μmol/L)	360.21 ± 88.49	335.59 ± 86.55	330.12 ± 93.54	338.71 ± 103.79	<0.05*
CysC (mg/mL)	0.95 ± 0.24	1.00 ± 0.32	1.05 ± 0.42	1.10 ± 0.48	<0.001**
TG (mmol/L)	1.89 (1.27, 3.05)	1.53 (1.08, 2.29)	1.53 (1.05, 2.27)	1.53(0.97, 2.33)	<0.001**
TC (mmol/L)	4.68 ± 1.87	4.45 ± 1.47	4.35 ± 1.22	4.37 ± 1.48	>0.05
HDL (mmol/L)	1.10 ± 0.30	1.15 ± 0.31	1.17 ± 0.35	1.20 ± 0.31	<0.05*
LDL (mmol/L)	2.77 ± 0.99	2.84 ± 1.03	2.75 ± 1.10	2.67 ± 1.05	>0.05
HbA1C (%)	9.41 ± 2.40	9.20 ± 2.49	9.37 ± 2.50	9.31 ± 2.55	>0.05
UAER (mg/24h)	11.51 (6.29, 31.07)	9.24 (4.86, 24.48)	11.18 (5.98, 45.81)	12.68 (6.21, 36.97)	<0.05*
FCP (ng/mL)	2.30 ± 1.30	2.02 ± 1.27	2.11 ± 1.22	2.07 ± 1.22	>0.05
DPN (%)	9.2%	18.8%	25.1%	45.4%	<0.001**

Values were expressed as mean ± SD for normally distributed data and median with interquartile range for non-normally distributed data, or n (%). Differences among the groups were analyzed by ANOVA for normally distributed values and by the Kruskal–Wallis test for nonparametric values. Pearson’s χ2 test was employed to analyze categorical data. *p<0.05, **p<0.001. BMI, body max index; SBP, systolic blood pressure; DBP, diastolic blood pressure; BUN, blood urea nitrogen; Cr, serum creatinine; UA, serum uric acid; CysC, serum cystatin C; TG, triglycerides; TC, total cholesterol; HDL, high-density lipoprotein; LDL, low-density lipoprotein; HbA1c, glycated hemoglobin; UAER, urine albumin excretion rate; FCP, fasting c-peptide; DPN, diabetic peripheral neuropathy.

### NCS parameters in various groups based on RFMI

NCS indexes in various groups based on RFMI are shown in **Table 3**. Individuals with lower RFMI had significantly impaired NCS indexes, e.g., mean motor nerve amplitude (P<0.001), motor nerve CV (P<0.05), sensory nerve amplitude (P<0.001) and sensory nerve CV (P<0.001).

**Table 3:** Nerve conduction of T2DM patients stratified by RFMI categories.

		RFMI				P
		>2.81 (n=238)	2.37-2.81 (n=239)	1.96-2.37 (n=231)	≤1.96 (n=240)	
Motor nerve	Amp (mV)	12.36 ± 2.64	11.81 ± 2.75	11.08 ± 3.02	10.54 ± 3.28	<0.001**
	CV(m/s)	48.80 ± 7.18	48.32 ± 5.92	46.99 ± 8.22	46.20 ± 8.66	<0.05*
Sensory nerve	Amp(mV)	12.54 ± 5.36	11.71 ± 5.35	10.64 ± 4.61	9.59 ± 4.88	<0.001**
	CV(m/s)	51.79 ± 6.34	50.40 ± 7.68	48.95 ± 9.70	46.16 ± 10.86	<0.001**

Amp, amplitude; CV, conduction velocity; *p<0.05, **p<0.001.

### Associations of NCS indexes with RFMI in T2DM

Pearson’s correlation analysis of RFMI and mean amplitudes/CVs of motor/sensory nerves was carried out, respectively. RFMI had significant positive correlations with mean motor nerve amplitude (r=0.244), motor nerve CV (r=0.146), sensory nerve amplitude (r=0.224) and sensory nerve CV (r=0.253), as shown in **Table 4** (all P<0.001).

**Table 4:** Pearson’s correlation analysis of nerve conduction with RFMI.

		r	*P*
Motor nerve	Amp	0.244	<0.001**
	CV	0.146	<0.001**
Sensory nerve	Amp	0.224	<0.001**
	CV	0.253	<0.001**

Amp, amplitude; CV, conduction velocity. **p<0.001.

### Risk factors for DPN

For logistic regression analysis, continuous variables underwent transformation into hierarchical variates based on the following intervals: duration of diabetes (≤10 years and > 10 years), BMI (≤24 kg/m^2^, 24–28 kg/m^2^ and >28 kg/m^2^), HbA1c (≤7% and >7%), RFMI (>2.81 cm²/m², 2.37-2.81 cm²/m², 1.96-2.37 cm²/m² and ≤1.96 cm²/m²).

Multivariable logistic regression analysis was carried out to determine risk factors for DPN. Patient features such as diabetes duration, BMI, HbA1c and RFMI were transformed into qualitative data and used as independent variables together with gender.

Diabetes duration above 10 years (OR=2.59, 95% CI1.86-3.60; P<0.001) independently predicted DPN. Additionally, low RFMI values of 1.96-2.37 cm²/m² (OR=3.57, 95% CI2.11-6.05; P<0.001) and ≤1.96 cm²/m² (OR=6.41, 95% CI3.83-10.72; P<0.001) increased the risk of DPN compared with higher levels (>2.81 cm²/m²). These data are summarized in **Table 5**.

**Table 5:** Risk factors of DPN in multivariate logistic regression.

Variables	Odd ratio (95% Cl)	*P*
RFMI		
>2.81	1(Ref.)	
2.37-2.81	1.73(0.98-3.05)	>0.05
1.96-2.37	3.57(2.11-6.05)	<0.001**
≤1.96	6.41 (3.83-10.72)	<0.001**
Duration		
≤10years	1(Ref.)	
>10years	2.59(1.86-3.60)	<0.001**

RFMI, rectus femoris mass index;95% CI, 95% confidential interval. *p< 0.05. **p<0.001.

Thus, ROC analysis was performed to assess the optimal cutoffs of T2DM duration and RFMI used jointly to detect DPN. Another multivariable logistic regression analysis was carried out with DPN as a dependent variate and T2DM duration and RFMI as independent variates. This approach enabled the assessment of the T2DM duration/RFMI combination for its predictive potential in DPN.

**Figure 1:**
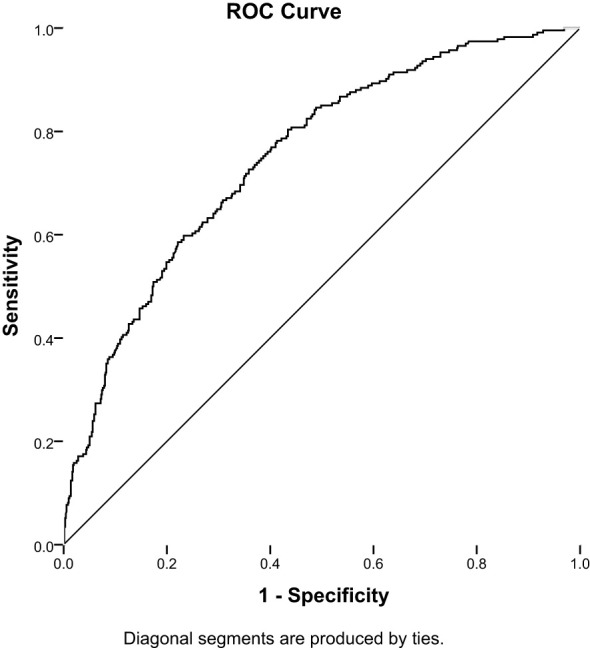
Receiver-operating characteristic (ROC) analysis of the duration of diabetes combined RFMI to predict DPN in T2DM patients (AUC = 0.75; 95% CI: 0.72–0.79; sensitivity,68.4%; specificity, 66.8%, P< 0.001).

The cutoffs for T2DM duration and RFMI were 7 years and 2.2 cm²/m², respectively; based on these values an AUC of 0.75 (95% CI: 0.72–0.79, P<0.001) was obtained, and sensitivity and specificity of 68.4% and 66.8%, respectively (**Figure 1**).

## Discussion

In this study, low RFMI was found to be associated with DPN in T2DM patients, corroborating previous reports. Henning Andersen and colleagues showed that diabetics have decreased lower limb muscle volume, which has a tight association with the degree of neuropathy (24). A systematic review and meta-analysis reported a significant correlation between sarcopenia and DPN (25). Christer SA and colleagues considered that impaired nerve function leads to denervation and muscle strength decrease in neuropathy cases (26). This work also showed age, gender, diabetes duration, BMI, BP control, renal function, TG and HDL were significantly different among groups categorized by RFMI. Anna Izzo and collaborators demonstrated sarcopenia has significant associations with age, gender, BMI, diabetes duration, glycemic control, microvascular/macrovascular complications, nutritional status and the intake of glucose-lowering products in T2DM patients (9).

Additionally, both the amplitude and CV of sensory/motor nerves declined with decreasing RFMI. Furthermore, RFMI and NCS indexes were positively correlated. These results showed that both demyelination and axonal degeneration of sensory/motor nerves are tightly correlated with sarcopenia in diabetics. Giorgio Orlando and colleagues showed that DPN affects the muscle by impairing motor nerve conduction (27). Andreassen and co-workers showed that DPN induces muscle atrophy and weakness via muscle denervation resulting from motor axon loss (10). Andersen and collaborators showed that muscle mass decline is associated with DPN severity and is more prominent distally (28). In the other hand, Zhang and colleagues showed elevated muscle mass improves the partial sensory/motor nerve conduction velocity (29). Nevertheless, the relationship between DPN and sarcopenia remains undefined.

Besides RFMI, risk factors for DPN also encompass diabetes duration based on logistic regression, consistent with previous findings (30, 31). The prevalence of DPN increases with diabetes duration, and studies have shown the risk of DPN shows a 7% increase with each additional year of diabetes course (32). A further study revealed DPN prevalence may reach 50% in patients with T2DM lasting more than 25 years (33). In this study, DPN cases showed elevated HbA1C compared with the non-DPN group (**Table 1**; P<0.001); however, in logistic regression, risk factors for DPN did not include HbA1C, which may be related to the fact that HbA1C only reflects the average blood glucose levels of patients in the first three months, which is not enough to reflect long-term blood glucose levels. The effect of gender on DPN is still unknown and most studies have not highlighted gender differences in DPN (34). Male diabetics were found to be more prone to DPN in some studies (35, 36). In the present study, the DPN group had a higher proportion of male, but in the regression analysis, there was no significant difference in gender between the two groups.

DPN incidence is elevated in diabetics, also representing a major cause of diabetes disability and even death (37). Sarcopenia also constitutes an important T2DM complication, and has recently attracted growing attention (13, 38). It increases the odds of adverse outcomes such as falls, frailty and death (39). According to available findings, DPN is correlated with muscle mass decrease, but a causal relationship between the two factors is not entirely clear. Meanwhile, no treatment slowing DPN development is currently available (40). Previous studies have shown that resistance exercise (41), endurance exercise and aerobic exercise (42) can effectively increase muscle mass in older adults. However, whether increasing muscle mass could delay the occurrence of DPN is unknown. Therefore, to avoid their interaction that may aggravate adverse effects in patients, it is recommended to conduct muscle mass measurement and DPN screening in early T2DM, and to intervene early in case of any abnormality.

In this study, ultrasound was utilized to evaluate muscle mass as a simple, non-radiative and highly operable tool, which can be easily applied in clinical and large-scale studies. Nevertheless, this study had some limitations. First, with a cross-sectional design, a causal relationship between RFMI and DPN could not be determined. Secondly, physical activity, which may be associated with muscle mass, was not assessed.

## Conclusion

Overall, low RFMI and DPN are significantly associated. This suggests T2DM duration and RFMI may help predict DPN prevalence. Specifically, patients with diabetes duration greater than 10 years and RFMI below 2.2 cm²/m² are closely associated with DPN.

## Data availability statement

The original contributions presented in the study are included in the article/supplementary material. Further inquiries can be directed to the corresponding author.

## Ethics statement

The studies involving human participants were reviewed and approved by Medical Ethics Committee, ShenZhen Hospital, Southern Medical University (NYSZYYEC20200030). The patients/participants provided their written informed consent to participate in this study.

## Author contributions

LW and XPL carried out the clinical studies, conducted the statistical analyses and wrote the first draft of the manuscript. HH and YW were involved in the interpretation of data. XXL and XZ contributed to the acquisition of data. LX designed the study, helped to review the manuscript and is the guarantor of this work. All authors edited, reviewed, and approved the final version of the manuscript.
